# Sirtuin1 Targeting Reverses Innate and Adaptive Immune Tolerance in Septic Mice

**DOI:** 10.1155/2018/2402593

**Published:** 2018-07-04

**Authors:** Ayana N. Martin, Martha Alexander-Miller, Barbara K. Yoza, Vidula Vachharajani, Charles E. McCall

**Affiliations:** ^1^Department of Molecular Medicine & Translational Science, Wake Forest School of Medicine, Winston-Salem, NC, USA; ^2^Department of Microbiology & Immunology, Wake Forest School of Medicine, Winston-Salem, NC, USA; ^3^Department of Surgery, Wake Forest School of Medicine, Winston-Salem, NC, USA; ^4^Department of Anesthesiology, Wake Forest School of Medicine, Winston-Salem, NC, USA; ^5^Department of Internal Medicine, Wake Forest School of Medicine, Winston-Salem, NC, USA

## Abstract

Resistance and tolerance to infection are two universal fitness and survival strategies used by inflammation and immunity in organisms and cells to guard homeostasis. During sepsis, however, both strategies fail, and animal and human victims often die from combined innate and adaptive immune suppression with persistent bacterial and viral infections. NAD^+^-sensing nuclear sirtuin1 (SIRT1) epigenetically guards immune and metabolic homeostasis during sepsis. Pharmacologically inhibiting SIRT1 deacetylase activity in septic mice reverses monocyte immune tolerance, clears infection, rebalances glycolysis and glucose oxidation, resolves organ dysfunction, and prevents most septic deaths. Whether SIRT1 inhibition during sepsis treatment concomitantly reverses innate and T cell antigen-specific immune tolerance is unknown. Here, we show that treating septic mice with a SIRT1 selective inhibitor concordantly reverses immune tolerance splenic dendritic and antigen-specific tolerance of splenic CD4+ and CD8+ T cells. SIRT1 inhibition also increases the ratio of IL12 p40+ and TNF*α* proinflammatory/immune to IL10 and TGF*β* anti-inflammatory/immune cytokines and decreases the ratio of CD4+ T_Reg_ repressor to CD4+ activator T cells. These findings support the unifying concept that nuclear NAD^+^ sensor SIRT1 broadly coordinates innate and adaptive immune reprogramming during sepsis and is a druggable immunometabolic enhancement target.

## 1. Introduction

A universal concept in evolutionary biology is that the inflammatory stress response protects homeostasis by *resistance or tolerance* [1, 2]. In sepsis extreme systemic inflammation [3], the high energy-demanding switch that promotes anabolic growth and differentiation of biosynthetic processes needed to *resist* invading microbes rapidly switches to *tolerance*, a very low energy state simulating suspended animation or severe starvation that invokes profound immune suppression of both innate and antigen-specific T cells; many sepsis victims perish from persistent infections associated with immune failure. Understanding how sepsis survivors “break” immune tolerance and its attendant profound innate and adaptive immune suppression to resolve the acute inflammatory response to infection may inform new ways to treat sepsis.

We pioneered the concept that NAD^+^ redox and intermediary metabolism sensors sirtuin1 (SIRT1) and sirtuin6 (SIRT6) epigenetically reprogram the universal attributes of resistance to tolerance in monocytes by shifting glycolysis and glucose oxidation high energy use to the low energy state lipolysis by generating silent heterochromatin at selective sets of immune and metabolism fueling gene sets (TNF*α*) [4, 5] and maintaining open euchromatin at reciprocally functioning gene sets [6–8]. We further showed that chronic NAD^+^ generation persistently promotes SIRT1 activation in monocytes to maintain immune tolerance in monocytes in mice and human sepsis [5]. Strikingly, inhibiting SIRT1 in septic mice reverses innate immune tolerance in monocytes and tolerance of microvascular leukocyte/endothelial adherence interactions and promotes survival [9]. Moreover, interferon gamma (IFN*γ*) administered in vivo or ex vivo to mouse or human monocytes obtained during sepsis reversed the phenotype of glycolysis deactivation and innate immune tolerance of monocytes [10].

Studies in mice and humans indicate that adaptive immunity T cells also become tolerant during sepsis, variously called “anergy” in CD4+ Th1 effector cells [11, 12] and “exhaustion” in CD8+ cytotoxic T cells [13, 14]. Mounting data support that metabolic substrate selection drives inflammation in innate and adaptive immune cell effector and repressor phenotypes [15–18]. Like innate immune monocytes, tolerant adaptive immune T cells are unable to mount a glycolysis-dependent response needed to anabolically fuel immune resistance responses following antigen-receptor stimulation, suggesting a common checkpoint for immune reprogramming [13]. Clinically, the emergence of tolerant innate and adaptive immune cells during sepsis also parallels elevations in CD4+ T regulatory repressor (T_Reg_) [19] cells, as well as the increased production of anti-inflammatory and antiglycolytic interleukin 10 (IL10) [20] and immune repressor transforming growth factor beta (TGF*β*) [21]. Increases in the ratio of immune repressor cell T_Reg_ and exhausted/anergic/tolerance phenotypes during sepsis mark the clinical state of chronic bacterial and recrudescent viral infections [22, 23]. However, whether innate and adaptive immunity and organ-specific tolerance states are under a unified control axis during sepsis is unknown [24].

SIRT1 is a nuclear NAD^+^ redox and metabolism sensor that acts epigenetically in most cells as a homeostasis guardian that coordinates immune and inflammation polarity in [5, 25–29]. SIRT1 reprogramming of immune polarity occurs in vitro in monocytes [4, 5, 30–32], macrophages [33, 34], and in CD4 and CD8 T cells and tolerogenic dendritic cells [35–40]. SIRT1 plays a key role in inducing CD11c tolerogenic dendritic cells in vivo in mice and informs CD4+ T effector Th1 and repressor CD4+ repressor cell and their respective link to cytokine production during endotoxin tolerance [36]. An in vivo link between innate and adaptive immune resistance and tolerance reprogramming during sepsis has never been reported to our knowledge. If this coupling occurs during sepsis, it would suggest that SIRT1 targeting of the global immune dysregulated state of innate and adaptive immune cells might inform a unified sepsis treatment approach for immune enhancement treatment of human sepsis.

Here, we report that therapeutic targeting of immune tolerant C57BL/6 septic mice 24 h after cecal ligation and puncture (CLP) with 10 mg/kg of EX-527, a SIRT1-selective inhibitory dose substantially increases survival [9], reverses innate and adaptive immune tolerance. EX-527 decreased the CD4+Foxp3+CTLA4+ T_Reg_ cell population able to express IL10 and TGF*β* repressor cytokines and increased the proportion of CD4+ T cells able to express interferon *γ*. EX-527 also reversed antigen-receptor-dependent tolerance among total splenocyte adaptive immunity populations. Concomitant with the switch away from adaptive immune cell tolerance toward the effector *resistance*, phenotype was reversal of splenic tolerogenic CD11c+ dendritic cells, as evidenced by increased interleukin 12 p40 (IL12 p40) and TNF*α* expression following nonspecific cell stimulation. Remarkably, as we had found for innate immune monocytes [9], SIRT1 inhibition significantly switched the adaptive immunity away from tolerance toward resistance within 6 h after a single dose of EX-527. This study is consistent with the unifying concept that a nuclear immunometabolic checkpoint controlled at least in part by SIRT1 directs innate and adaptive immune reprogramming during sepsis and informs molecular-based immune axis targeting.

## 2. Materials and Methods

### 2.1. Mice

This study was approved by the Institutional Animal Care and Use Committee of the Wake Forest School of Medicine according to NIH guidelines. 6–8-week-old male WT mice (C57Bl/6) from Jackson Laboratory (Bar Harbor, ME, USA) were randomized into Sham, CLP, or CLP + EX-527 groups, with 5 mice/experimental group. The experimental protocol for this study was used precisely as previously reported for EX-527 to test its effect on innate immunity, vascular and microvascular function, and survival [5]. The present mice were used to compare previous studies of innate immunity with this focused study of innate and adaptive immunity in concert.

### 2.2. CLP Sepsis Model

Cecal ligation and puncture (CLP) has been standardized in our sepsis model in C57Bl6 mice [5]. Briefly, the cecum was externalized from the peritoneal cavity, ligated, and perforated twice with a 22-gauge needle, which induces a ~60% 14 d mortality rate. For the sham surgery, the cecum was externalized and returned to the cavity. Fluid resuscitation (1 mL normal saline) was administered s.c. after surgery. No antibiotics were given.

### 2.3. SIRT1 Targeting Treatment Design

Treatment protocol was followed exactly as reported in the SIRT1 study of monocytes and sepsis outcome [5]. Briefly, 10 mg/kg (4 mL/kg) of EX-527 (made in DMSO and delivered in normal saline) was injected i.p. 24 h postsurgery in CLP animals; untreated CLP and Sham control animals received equivalent volume of DMSO (4 mL/kg) in normal saline at 24 h postsurgery of about 1 *μ*M, as a small molecule broadly distributes in tissue and has no known off target effects. EX-527 (obtained from Sigma Chemicals, St. Louis, MO) is a potent and selective inhibitor of SIRT1 activity [41]. It binds in the catalytic cleft of SIRT1, displacing the NAD^+^ and forcing the cofactor into an extended conformation, thus sterically preventing substrate binding in the catalytic domain. It is 200–500-fold more selective for SIRT1 than for SIRT2, SIRT3, or SIRT6, has a half-life of 2 h, and is active in <100 nM concentrations [42].

In selecting the time for EX-527 i.p. administration for this study, we were guided by our previous report that microvascular inflammation tolerance in vivo is fully established by 24 h after CLP. We further reported that 6 h posttreatment time point (30 h after CLP) detects EX-527-dependent tolerance reversal in monocyte cytokine production and in microvascular leukocyte-endothelial adhesion [5]. Accordingly, this study focused on the 24 h sepsis post-CLP as treatment time and the previously used 6 h posttreatment time point for analyses of innate and adaptive immunity, using isolated splenocytes. Markedly increased survival after EX-527 occurs under these same conditions.

### 2.4. Splenocyte Isolation and Flow Cytometry

Splenocytes were harvested as reported [9], using 500 *μ*L Collagenase D solution at a concentration of 1 mg/mL, which was scissor cut and passed through a 70 *μ*m cell suspensions of 1 × 10^7^ cells/mL in Flow Cytometry Staining Buffer (PBS with 5% FBS). Cells were run on the BD FACSCanto II and analyzed using FlowJo v10 for assessing scatter, frequency, cell number, and mean fluorescent intensity per cell. For 6 h and for 12 h postsepsis treatment analyses, sham versus CLP animals were compared. For 24 h post-CLP treatment, sham versus CLP versus CLP + EX-527 animals were compared after 6 h (30 h post-CLP).

### 2.5. Antibodies Used in Flow Cytometry

Cell surface antigens were stained first. The dendritic cell antibody panel quantified the following: CD11c-APC-eFluor780, CD80-PE-Cy7, CD86-FITC, and CD40-eFluor450 (all mAbs are from eBioscience now Thermo Fisher, Waltham, MA). The CD4+ T_Reg_ cell panel included the following: CD3*ε*-PE, CD4-PE-Cy7, and CD25-PerCP-Cy5.5s (all mAbs are from eBioscience). For T_Reg_ intracellular staining, cells were fixed and permeabilized with the one step eBioscience Fix-Perm Foxp3 Buffer Staining Kit (eBioscience). The panel included Foxp3-eFluor450 and CTLA4-APC (mAbs are from eBioscience). Intracellular staining was also used to detect TGF*β*-PE, IL12 p40-PE, IL10-APC, and IFN*γ*-APC production (mAbs are from eBioscience) after ex vivo nonspecific antigen stimulation with a Leukocyte Activation Cocktail with GolgiPlug (BD Biosciences) for flow cytometry. Mouse INF*γ* Single-Color ELISPOT to determine antigen-specific response of T cells was from Cellular Technology Limited (CTL), Cleveland, OH. For attempting to assess SIRT1 expression by flow cytometry, we used antibodies from Santa Cruz and Abcam.

### 2.6. Data Analysis

All data were analyzed using GraphPad Prism 6.0 (GraphPad Software, La Jolla, CA, USA). Our studies are powered at 5–7 animals per group per 2 experiments, but the numbers are increased as needed based on variability. For analyses between two population means, we used unpaired, two-tailed Student's *t*-test analyses. Groups of more than three comparisons were analyzed using one-way ANOVA, followed by Tukey's post hoc *t*-test. Significance is indicated with an asterisk indicating ^∗^ *p* < 0.05. Error bars represent ±SEM. In the figures, all values are depicted with the number of animals in the experimental conditions along with single asterisks indicating our significance threshold of *p* < 0.05 to make it easier to follow. The precise value can be found in the text. Trends not reaching the *p* < 0.05 can be appreciated by scatter box depictions.

## 3. Results

### 3.1. Total CD4+ T Cells, but Not Total CD8+ T Cells, Decrease in the Spleen during Early Sepsis

**Figure 1:**
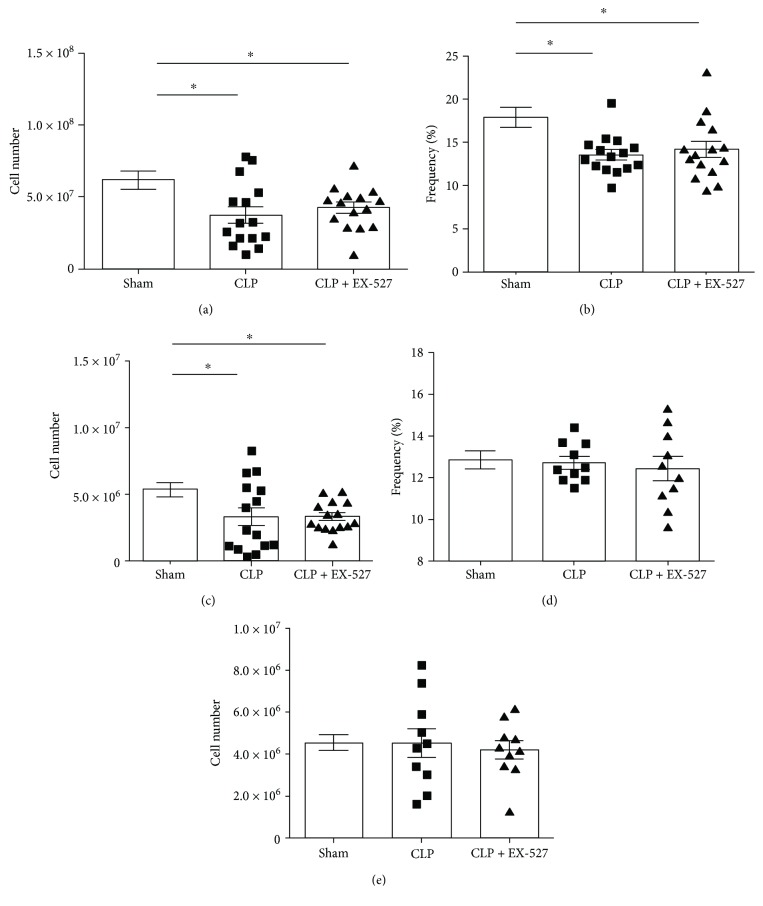
SIRT1 inhibition has no effect on frequency and number changes in total splenocyte and CD4+ T cells during sepsis-induced immunosuppression. All data presented as sham versus CLP versus CLP + EX-527. (a) Cumulative results of total splenocytes presented as total cell number. (b, c) Cumulative results from surface CD4 presented as (b) frequency of total splenocytes (%) and (c) absolute cell count. (d, e) Cumulative results from surface CD8 presented as (d) frequency of total splenocytes (%) and (e) absolute cell count. Data are representative of three independent analyses with a total of 5 mice in each group. Data are expressed as mean ± SEM. *n* = 15 mice/group. ^∗^ *p* < 0.05.

T_Reg_ cells can be found in both CD4+ and CD8+ T cell populations [43]; therefore, we first determined whether the CD4+ and/or CD8+ T cell populations were impacted by early sepsis. Splenocytes isolated from the spleens of sham and CLP mice were stained with anti-CD4 and anti-CD8 antibodies. Total splenocyte counts decreased in the CLP mice, compared to the sham control (Figure 1(a); sham = 6.18 × 10^7^ ± 6.35 × 10^6^ cells, CLP = 3.75 × 10^7^ ± 5.84 × 10^6^ cells; *p* = 0.0086). At 30 h post-CLP, the frequency of CD4+ T cells in CLP mice significantly decreased as compared to sham (Figure 1(b); sham = 17.94 ± 1.98%, CLP = 13.57 ± 0.59%; *p* = 0.0025). This difference also translated to the absolute cell count (Figure 1(c); ham = 5.35 × 10^6^ ± 5.40 × 10^5^ cells, CLP = 3.35 × 10^6^ ± 6.77 × 10^5^ cells; *p* = 0.0285). In contrast to the sepsis-induced changes in CD4+ T cells at 30 h, the frequency and absolute cell numbers of CD8+ T cells were similar between sham and CLP (Figures 1(d) and 1(e); *p* = 0.79 and *p* = 0.99, resp.). SIRT1 inhibition with EX-527 had no effect on the diminished CD4+ T cell population in frequency or absolute cell count compared to CLP treatment (Figures 1(b) and 1(c); *p* = 0.55 and *p* = 0.98, resp.). CD8+ T cells also showed no changes in response to SIRT1 inhibition in frequency or absolute cell count (Figures 1(d) and 1(e); *p* = 0.68 and *p* = 0.69, resp.). Taken together, these data suggest that sepsis differentially affects CD4+ versus CD8+ T cells during the early immunosuppression. These data are similar to other reports on sepsis-induced apoptosis dominance in CD4+ T cells and dendritic cells [44].

### 3.2. SIRT1 Inhibition Reverses CD4+ T Cell Cytokine Polarity and Tolerance of Total T Cell IFN*γ* Expression after Antigen-Receptor Stimulation

**Figure 2:**
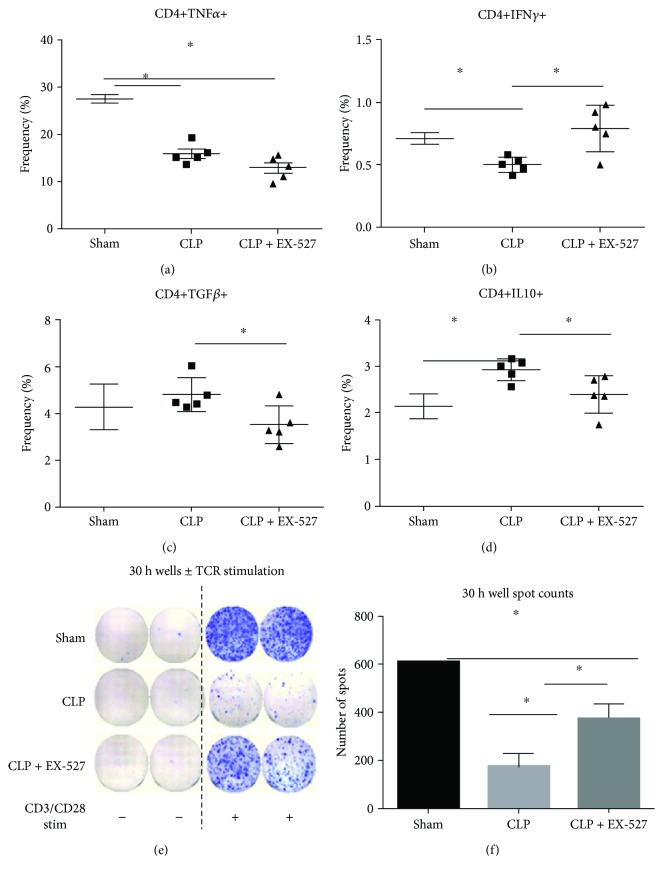
CD4+ T cell-associated cytokines are reprogrammed in a SIRT1-dependent manner. Cumulative scatterplot data of proinflammatory (a) CD4+TNF*α*+ T cells and (b) CD4+IFN*γ*+ T cells presented as frequency of CD4+ T cells in sham versus CLP versus CLP + EX-527. Cumulative scatterplot data of anti-inflammatory (c) CD4+TGF*β*+ T cells and (d) CD4+IL10+ T cells presented as frequency of CD4+ T cells in sham versus CLP versus CLP + EX-527. (e) Representative ELISPOT data of IFN*γ* producing T cells ± restimulation from sham versus CLP versus CLP + EX-527 mice. (f) Cumulative results presented as spots counted per well from sham versus CLP versus CLP + EX-527, based on cells seeded at 3 × 10^5^ cells/well. Data are expressed as mean ± SEM. (a–d) *n* = 5 mice/group; (e, f) *n* = 3 mice/group. ^∗^ *p* < 0.05.

To build upon our unifying concept of SIRT1 as a master homeostat of immune system function, we tested cytokine secretion in the spleen as ex vivo biomarkers of a functional switch in immune function. To do this, splenocytes were collected at 30 h from sham, CLP, and CLP + EX-527 mice and treated with phorbol myristate acetate (PMA) + ionomycin cocktail to induce cytokine production, while preventing release with GolgiPlug in order to detect by flow cytometry. In Figure 2, we show a shift away from proimmune CD4+ T cell responses toward anti-inflammatory responses during sepsis that support a suppressive CD4+-adaptive immune response. The frequency of TNF*α*, a major proinflammatory and immune activator, is decreased in septic mice at 30 h compared to sham (Figure 2(a); sham = 27.44 ± 0.86%, CLP = 15.84 ± 0.94%; *p* = 1.73 × 10^−5^). Similarly, the frequency of proimmune mediator IFN*γ* also decreased in septic mice at 30 h compared to sham (Figure 2(b); sham = 0.78 ± 0.02%, CLP = 0.5 ± 0.03%; *p* = 0.0003). Concomitant with the decrease in CD4+ T cell-derived proinflammatory cytokine production, the frequency of key repressor IL10 was significantly increased in septic mice at 30 h compared to sham (Figure 2(d); sham = 2.14 ± 0.12%, CLP = 2.92 ± 0.1%; *p* = 0.0011). While TGF*β* was not significantly increased during sepsis (Figure 2(c)), EX-527 significantly decreased TGF*β* (Figure 2(c)) and IL10 (Figure 2(d)) frequency compared to nontreated CLP mice (*p* = 0.029 and *p* = 0.035, resp.). Importantly and concurrent with the shift away from CD4+ anti-inflammatory cytokine production, SIRT1 inhibition increases in the frequency of IFN*γ* cytokine production in CD4+ T cells, compared with CLP (Figure 2(b); CLP = 0.5 ± 0.03%, CLP + EX-527 = 0.79 ± 0.08%; *p* = 0.011). These findings support the reversal of CD4+ Th1 cell anergy during SIRT1 inhibition.

To assess the function of the antigen-receptor following SIRT1 treatment during sepsis immune tolerance, we measured IFN*γ* production from total T cell antigen-receptor-stimulated T cells (including CD4+ and CD8+). Splenocytes isolated from sham, CLP, and CLP + EX-527 mice were restimulated overnight with *α*-CD3/*α*-CD28 at 500 ng/mL and 5 *μ*g/mL, respectively. IFN*γ* production was quantified using ELISPOT by counting the number of spots with immunoprecipitated detection antibody (Figure 2(e)).  Figure 2(f) demonstrates that CD3/CD28 antigen-receptor stimulation is unable to increase IFN*γ* production during sepsis (sham = 610 ± 2.5 spots per 3 × 10^5^ cells, CLP = 173 ± 56.4 spots per 3 × 10^5^cells; *p* = 0.0011), supporting antigen-specific receptor “tolerance” during sepsis. This in vivo assessment aligns with in vitro studies showing SIRT1 as a key promoter of antigen-dependent tolerance of CD4+ and CD8+ T cells [30]; SIRT1 is also a key contributor to tolerance monocytes in vitro and in mouse sepsis [4, 5, 7]. In this study, EX-527 selective SIRT1 inhibitor increased IFN*γ* secretion after TCR stimulation compared to CLP (Figure 2(f); CLP = 173 ± 56.4 spots per 3 × 10^5^ cells; CLP + EX-527 = 378 ± 57.2 spots per 3 × 10^5^ cells; *p* = 0.034). Taken together with published data and our findings, these findings support that SIRT1 reverses CD4+ tolerance and CD8+ T cell exhaustion and broadly promotes T cell homeostasis. We were unable to detect SIRT1 in any spleen cell by flow cytometry, leaving the possibility that cell autonomous or nonautonomous SIRT1-dependent cross-talk reprograms the CD4+ and CD8+ homeostat.

### 3.3. SIRT1 Inhibition Decreases CD4+Foxp3+ T_Reg_ Repressor Frequency

**Figure 3:**
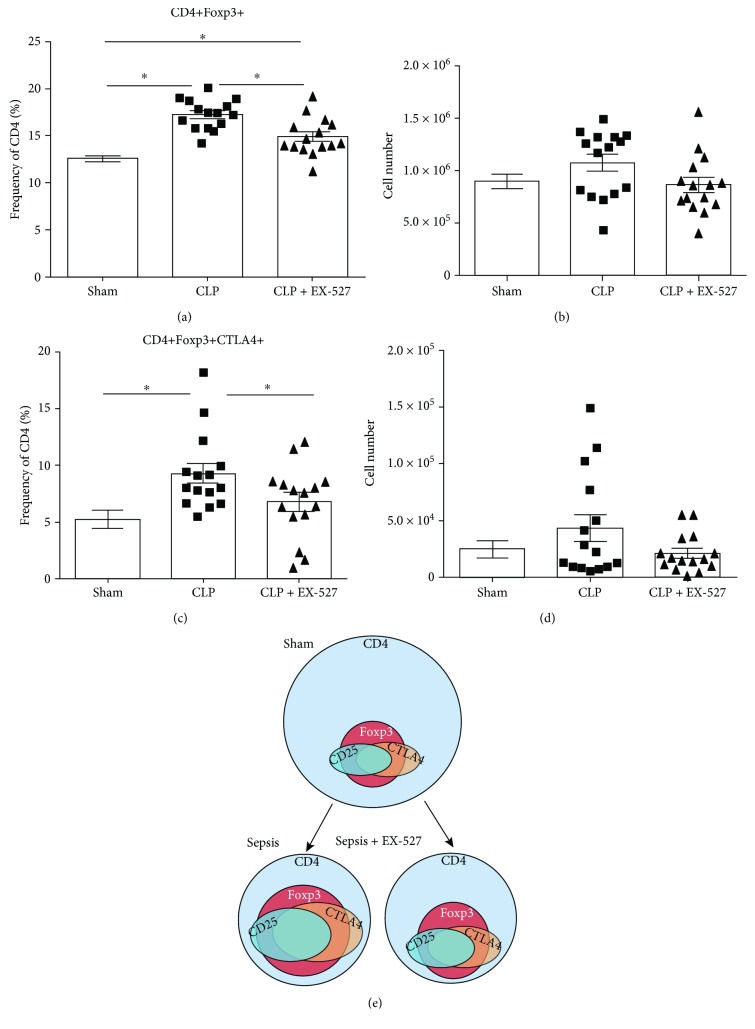
The frequency of CD4+Foxp3+ T_Reg_ cells reversibly increases in sepsis with SIRT1 inhibition. Cumulative results of CD4+Foxp3+ T_Reg_ cells presented as (a) frequency of CD4 (%) and (b) absolute cell count. Cumulative results of CD4+Foxp3+CTLA4+ T_Reg_ cells presented as (c) frequency of CD4 (%) and (d) absolute cell count. (e) Representative figure of the ratio changes in population size of CD4+ T cells and CD4+ T_Reg_ subpopulation. Markers are not representative of surface versus intracellular location. Data are representative of three independent analyses with a total of 5 mice in each group. Data are expressed as mean ± SEM. *n* = 15 mice/group. ^∗^ *p* < 0.05.

SIRT1 may also promote the repressor phenotype of CD4+Fox3p+ T_Reg_ cells, which are known to increase during sepsis [19, 45, 46]. We next tested whether the CD4+ T_Reg_ cells increase in early sepsis of our model and might contribute along with CD4+ T cell anergy to immune suppression. We first determined the CD4+ subpopulation of T_Reg_ cells exclusively, as there were no changes in the CD8+ T cell population at the 30 h postsepsis time point of our study on innate and adaptive immunity coordination. CD4+ T_Reg_ cells were defined by Foxp3 transcription factor, the master regulator of T_Reg_ cell lineage [47]. CD4+Foxp3+ T_Reg_ cells from CLP mice significantly increased to 17.26 ± 0.41% from the 12.56 ± 0.34% CD4+Foxp3+ T_Reg_ cells from sham mice (Figure 3(a), *p* = 1.46 × 10^−9^). The absolute cell count was not statistically significant (Figure 3(b); sham = 8.97 × 10^5^ ± 7.04 × 10^4^ cells, CLP = 1.07 × 10^6^ ± 8.23 × 10^4^ cells; *p* = 0.11). These data suggest reciprocal changes CD4+ Th1 and T_Reg_ repressor cells occur in the spleen in septic mice and are directly or indirectly coordinated by SIRT1. They further support SIRT1 as a molecular target for deprogramming tolerance and immune repression during sepsis.

We previously demonstrated disrupting SIRT1 24 h after sepsis promotes immunometabolic competence in monocytes within 6 h and markedly improves 7 day survival in septic mice [5, 9, 32]. Surprisingly, SIRT1 inhibition by a single i.p. dose of EX-527 also shifts adaptive immunity within 6 h of treatment (Figure 3(c)), as supported by the significantly reduced CD4+Fox3 T_Reg_ CTLA+ frequency following EX-527 treatment of septic mice (CLP = 11.37 ± 0.79%, CLP + EX-527 = 8.76 ± 0.63; *p* = 0.0156).

To better characterize the biological impact of the enhanced T_Reg_ cell proportions during sepsis-induced polarization, CTLA4 expression was measured as a marker for the immune repressor property of T_Reg_ cells [48]. Figure 3(c) shows that CD4+Foxp3+CTLA4+ T_Reg_ cells increase in frequency during sepsis as compared to sham (sham = 5.26 ± 0.81%, CLP = 9.31 ± 0.88%; *p* = 0.002) and that SIRT1 inhibition significantly reduces CD4+Foxp3+CTLA4+ T_Reg_ cell subpopulations (Figure 3(c)). The absolute cell counts of all the measured T_Reg_ subpopulations also decreased, but without statistical significance (Figure 3(d)).

Taken together, the changes in CD4+Foxp3+ and CD4+Foxp3+CTLA4+ T_Reg_ cells suggest that SIRT1 inhibition selectively reprograms the balance of CD4+ T cells to favor improved CD4+ T cell effector immune functions. This switch occurs without detectable changes in the total despite splenocyte population after a single dose of EX-527.  Figure 3(e) summarizes CD4+ T_Reg_ cell population and immune functional changes. The representative figure shown highlights the ratio changes in population size of CD4+ T cells and CD4+ T_Reg_ subpopulation (note: markers are not representative of surface versus intracellular location). During sepsis, the total CD4+ T cell population significantly decreases, while the CD4+ T_Reg_ cell subpopulation significantly increases, thus shifting the balance in favor of immune suppression. SIRT1 inhibition reduces the CD4+ T_Reg_ cell subpopulation, without increasing the CD4+ T cell population. This subpopulation reprogramming may restore adaptive immune homeostasis.

### 3.4. SIRT1 Inhibition Shifts Reprogramming of DC CD80 and CD86 Expression

Recently published data [36] clearly showed that SIRT1 plays an obligated role in supporting the function of tolerogenic dendritic cells in C57BL6 mice and their control of the CD4+ T effector and T repressor cell polarity. That study used genetic and pharmacologic analysis with EX-527; however, it did not investigate sepsis, which does not lend itself to genetic approaches to follow resistance and tolerance reprogramming kinetically. Mature DCs activate T cells through costimulatory molecules CD80 and CD86 and then are reprogrammed during sepsis to a tolerogenic state [49, 50]. CD80 may promote and CD86 may inhibit T_Reg_ cell proliferation [51–53].

**Figure 4:**
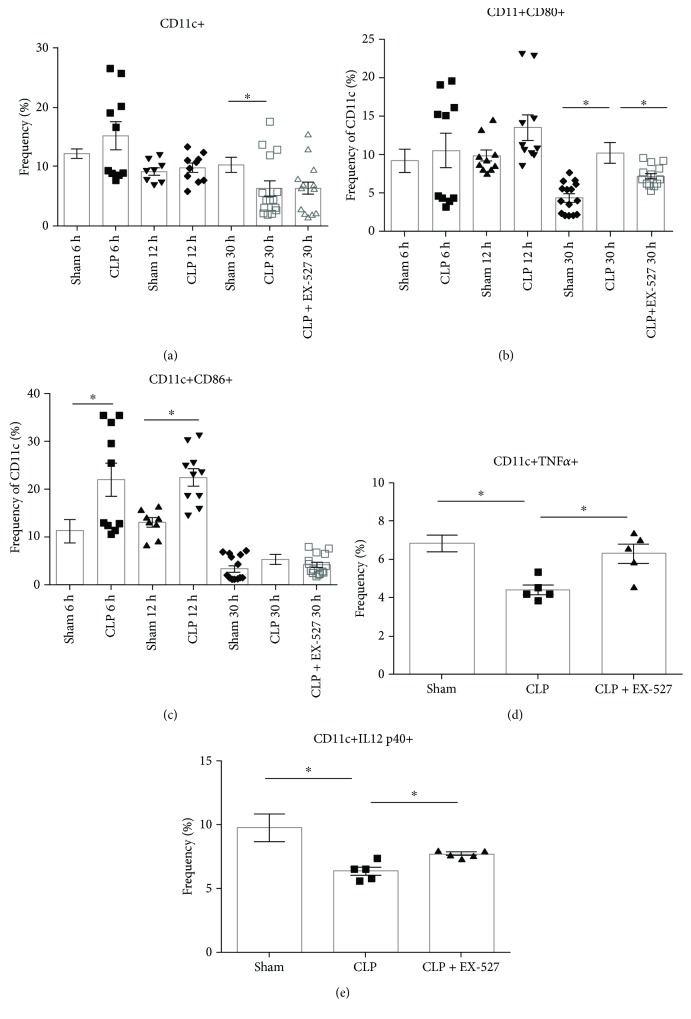
Effects of sepsis and SIRT1 inhibition on CD11c+ DC costimulatory markers. (a) Cumulative results of CD11c+ DCs presented as frequency of total splenocytes. (b, c) Cumulative data of CD11c+CD80+ DCs (b) and CD11c+CD86+ DCs (c) presented as frequency of CD11c. (d, e) Cumulative results of CD11c+ DC production of proinflammatory TNF*α* (d) and IL12 p40 (e) cytokines presented as frequency of CD11c. (a–c) Data are representative of two independent analyses with a total of 5 mice in each group. (d, e) Data are a single preliminary analysis with a total of 5 mice in each group. Data are expressed as mean ± SEM. ^∗^ *p* < 0.05.

To identify splenic DCs and test their role in sepsis reprogramming of CD4+ T cells, isolated splenocytes from sham, CLP, and CLP + EX-527 mice were stained with anti-CD11c, anti-CD80, and anti-CD86 antibodies. Because CD11c+ DCs are innate immune cells that communicate with adaptive immune cells, CD11c+ DCs were assessed at 6 h, 12 h, and 30 h, including a 24 h CLP with and without EX-527 treatment to determine kinetics. Consistent with splenocyte apoptosis [54], CD11c+ DCs decreased at 30 h post-CLP (Figure 4(a); sham = 10.3 ± 1.29%, CLP = 6.33 ± 1.31%; *p* = 0.0395). The differential changes in CD80 and CD86 expression on CD11c+ DCs during the early and late phases of our sepsis model suggest that CD80+ and CD86+ are differentially modulated due to sepsis, so we determined whether SIRT1 inhibition balances this pattern. SIRT1 inhibition by EX-527 did not alter CD11c+ percentages in the spleen (Figure 4(a)), but the frequency of CD11c+CD80+ DCs was reduced compared to CLP (Figure 4(b)). In contrast, SIRT1 inhibition had no significant effect on the frequency or fold change of CD11c+CD86+ dendritic cells compared to CLP (Figure 4(c); *p* = 0.37). These data suggest that SIRT1 inhibition during sepsis may promote dendritic cell homeostasis. However, as with T cells, we were unable to track expression of SIRT1 protein in dendritic cells by flow cytometry.

### 3.5. SIRT1 Inhibition Repolarizes the CD11c+ DC Tolerogenic and Activator Axis

To further define functional changes in CD11c+ DCs that might influence T cell programming, pro- and anti-inflammatory cytokines were measured in splenic DCs. TNF*α* and IL12 p40 productions were measured to determine DC proinflammatory or activator responses, and IL10 and TGF*β* were measured to determine DC anti-inflammatory or tolerogenic responses. Figures 4 and 4 shows that both TNF*α* and IL12 p40 proinflammatory cytokines significantly decrease in CLP mice during sepsis tolerance (30 h) compared to sham (*p* = 0.0013 and *p* = 0.015, resp.). SIRT1 inhibition increases TNF*α* and IL12 proinflammatory cytokine production in CD11c+ DCs to a level similar to sham (CLP versus CLP + EX-527; *p* = 0.0093 and *p* = 0.0034, resp.). Levels of IL10 and TGF*β* in CD11c+ DCs at this time point were negligible (data not shown). Taken together, these data are compatible with the known function of SIRT1-dependent control of tolerogenic CD11c+ DC cell regulator of SIRT1-dependent adaptive immune polarity in the spleen of septic mice. These findings are consistent with our previous discovery that SIRT1 shifts innate immune monocytes from the tolerant phenotype to the activator phenotype during sepsis [5] and that the SIRT1 checkpoint may broadly influence reprogramming of innate and adaptive immunity reprograming during life-threatening sepsis.

## 4. Discussion

This study supports concordant control of innate and adaptive immunity axes during sepsis. The evidence supporting for this broad unifying pathophysiology concept includes that (1) NAD^+^-dependent and metabolic sensor SIRT1, as evidenced by the fact that a selective inhibitor EX-527 coordinates innate and adaptive immunity in septic mice, reprograms splenic CD11c+ DC subpopulation frequency from a tolerogenic to activator phenotype with increased expression of proimmune IL12 p40 and TNF*α*; (2) treatment increases the proportion of IFN*γ* effector CD4+ T cell subpopulations and decreases the frequency of CD4+Foxp3+ T_Reg_ CTLA4+ repressor cell proportions and decreases the frequency of CD4+ T_Reg_ cell expression of immune repressor cytokines TGF*β* and IL10; (3) treatment reverses antigen-receptor immune tolerance among all splenocytes, as evidenced by increased interferon *γ* immune expression; (4) and effects of a single dose of SIRT1-selective inhibitor EX-527 administered 24 h post-CLP persist for at least for 48 h in the CD4+ T population (Supplemental Figure S1/2).

Sepsis broadly dysregulates immune and organ cells and organism metabolism. Others [55–57] and we [4, 5, 9, 31] have shown that nuclear SIRT1, SIRT2, SIRT6, and mitochondrial SIRT3 drive immunometabolic reprogramming mechanistically by deacetylating key immune transcription factors, histone structural mediator regulatory factors, and methylating DNA [58]. SIRT1, lying proximal in immunometabolic signaling, deacetylates and inactivates NF-*κ*B p65 proimmune transcription factor function and supports a shift to supporting NF-*κ*B RelB immune repressor functions by chromatin structural modifications [59]. SIRT1, SIRT2, SIRT6, SIRT3, and NF-*κ*B, among other transcription and histone modifiers, ubiquitously control innate immune neutrophils, monocytes, and dendritic cells, as well as adaptive immunity CD4+CD8 T cells [30, 57, 60–63].

SIRT1, SIRT6, and SIRT3 are key redox sensors that inform glucose metabolism for use in effector immune anabolic signaling and its transition to repressed glucose use in increased activation of the lipolysis-fueled mitochondrial catabolism axis of resistance and tolerance [64, 65]. Glucose fueling directs proinflammatory responses and immune anabolic resistance mechanisms of both innate and adaptive immune cells [65–68]. In contrast, anti-inflammatory responses or immune tolerance, which is also marked by immune repressor T_Reg_ cells, mostly use fatty acids for oxidative phosphorylation [31]. The importance for this study is that SIRT1 lies proximal to expression and functions of both nuclear SIRT6 and mitochondrial SIRT3 [65, 69–71] which form a key homeostasis regulatory node. This node and its downstream connections help coordinate anabolic and catabolic energy delivery to both immune and organ cells under stress. Inhibiting SIRT1 in septic mice increases glucose oxidation in splenocytes, while enhancing the innate immune response [4, 9, 32]. The importance for interpreting this study is that innate immune reprogramming of monocytes in septic mice occurs at the same time which dendritic cells and T cells reprogrammed from tolerance to resistance pathways in this study, as previously published [9]. Since SIRT1 lies proximal to altered immunometabolic programming, what lies proximal to the SIRT axis? Redox control of SIRT1 cysteine thiols also informs its deacetylase activity in cells under stress, which directs lipolysis versus lipogenesis [72–74]. We recently found that the direct and reversible oxidative state of cysteine thiol 144 on SIRT6 informs its glucose homeostat property [30], a critical pivot for substrate switching between glucose driven resistance and fatty acid driven tolerance in septic mice. Thus, mounting data suggest that redox oxidation and reduction switching drive fuel selection among immune cells and may occupy a more fundamental site for inflammation and immune regulation in sepsis. Supporting this are our recent findings that mitochondrial SIRT4 counters the redox regulation of SIRT1, SIRT6, and SIRT3 by increasing glycolysis and its support of glucose oxidation [75].

This study has important limitations. One is that we were unable to pin point in which immune cell(s) SIRT1 might be primarily directing immune axis polarity shifting among the cell types investigated in this study, since our antibodies were unable to detect SIRT1 in Western blot of histochemical analyses. The most clear example of a potential site from in vivo study was the recent report using pharmacologic and genetic deletion and addition analyses in C57BL6 mice and endotoxin treatment (not sepsis), which clearly linked SIRT1 in CD11c dendritic cells to reprogram the axis between CD4+ Th1 cells and CD4+ T_Reg_ repressor cells [36]; these results parallel what may be occurring in this sepsis in this study, and mechanistically this path requires deacetylation of NF-*κ*B p64 [5]. However, SIRT1 and other nuclear SIRTs can broadly act on innate and adaptive cells in a cell autonomous paths [76]. The second limitation is that genetic analysis is not yet possible in studying tolerance kinetically and investigations like this one must rely on drug targeting and the possibility of off target effects. Although EX-527 is a highly selective SIRT1 inhibitor at the dose used in this study [41], it may have unrecognized off target effects. Moreover, we also did not define how long the effects on SIRT1 on reversing global immunity might last, an important issue in translating this concept to human sepsis. The third limitation relates to our recent discovery that SIRT2 and not SIRT1 regulates the immune homeostasis node in obese septic mice [55], lending support to the concept that redox regulation informs the SIRT family for homeostasis regulation depending on the cell type or organism energy phenotype.

**Figure 5:**
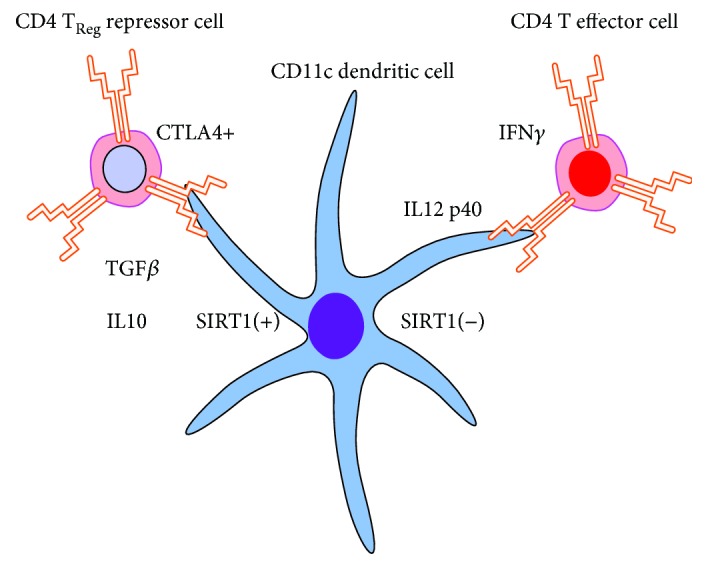
Effects of SIRT1 inhibition on dendritic and CD4 effector and repressor immunity during sepsis. SIRT1 increases during the immunometabolic stage of sepsis coordinates innate tolerogenic dendritic cell and CD4 T cell repressor and immune inhibitory IL10 and TGF*β* cytokines. SIRT1 therapeutic inhibition reverses both the innate to adaptive immune CD4 effector cell interactions and effector immune cytokines (e.g., IL12 p40 and interferon *γ* levels). It is unclear in which cell or cell nuclear SIRT1 is acting to guard resistance and tolerant sepsis inflammatory responses.

In conclusion, this study shows for the first time in a lethal model of sepsis in mice that a regulatory node controlled by a selective SIRT1 inhibitor known to improved sepsis survival [9] coordinates innate and adaptive immune cell polarity.  Figure 5 schematically demonstrates this discovery. A plausible common mechanistic explanation for SIRT1 bridging of both innate and adaptive immunity is its known effects of controlling immunometabolism reprogramming by epigenetically switching immune resistance mechanisms to immune tolerance mechanisms. This broad-based homeostasis axis is likely multimechanistic and not explained by targeted mechanistic studies so popular with reductionist biomedical investigations. However, it may open the way to better understand sepsis resolution and it informs broad-based immune targeting as potential sepsis treatment.

## Data Availability

The data used to support the findings of this study are available from the corresponding author upon request.

## Abbreviations

EX-527: Selective SIRT1 inhibitor
CLP: Cecal ligation and puncture
Foxp3: Transcription factor
SIRT: Sirtuin
T_Reg_: Regulatory T cell.

## Supplementary Materials

Supplementary 1 Figure 1: representative flow cytometry data of T cell subpopulations. Splenocytes from sham, sepsis (CLP), and septic mice treated with EX-527 (CLP + EX-527) were analyzed by FACS. Representative FACS data of CD8+ versus CD4+ T cells (A) and Foxp3+ versus CD4+ T cells (B). Gated cells are CD4+Foxp3+. Representative FACS data of CTLA4+ versus Foxp3+ T cells (C). Cells are gated on CD4+. Data are representative of three independent analyses; *n* = 15 mice/group.

Supplementary 2 Figure 2: the decrease in CD4+Foxp3+ T_Reg_ cells is maintained with SIRT1 inhibition at 48 h and the effect of SIRT1 inhibition on CD4+ reprogramming. FACS analysis CD4+ T cells (A) and CD4+Foxp3+ cells (B) at 12 (*n* = 5 mice/group), 30 (*n* = 15 mice/group), or 48 h (*n* = 5 mice/group) from sham, sepsis (CLP), and septic mice treated with EX-527 (CLP + EX-527). Data expressed as mean ± SEM. (C) Graphic representation of SIRT1 reprogramming CD4+ cells during sepsis.
