# Supplementary figures and images for: Sirtuin1 Targeting Reverses Innate and Adaptive Immune Tolerance in Septic Mice
Source: J Immunol Res. 2018 Jul 4;2018:2402593. doi: 10.1155/2018/2402593 (PMC6057336; doi:10.1155/2018/2402593)

## Slide 1
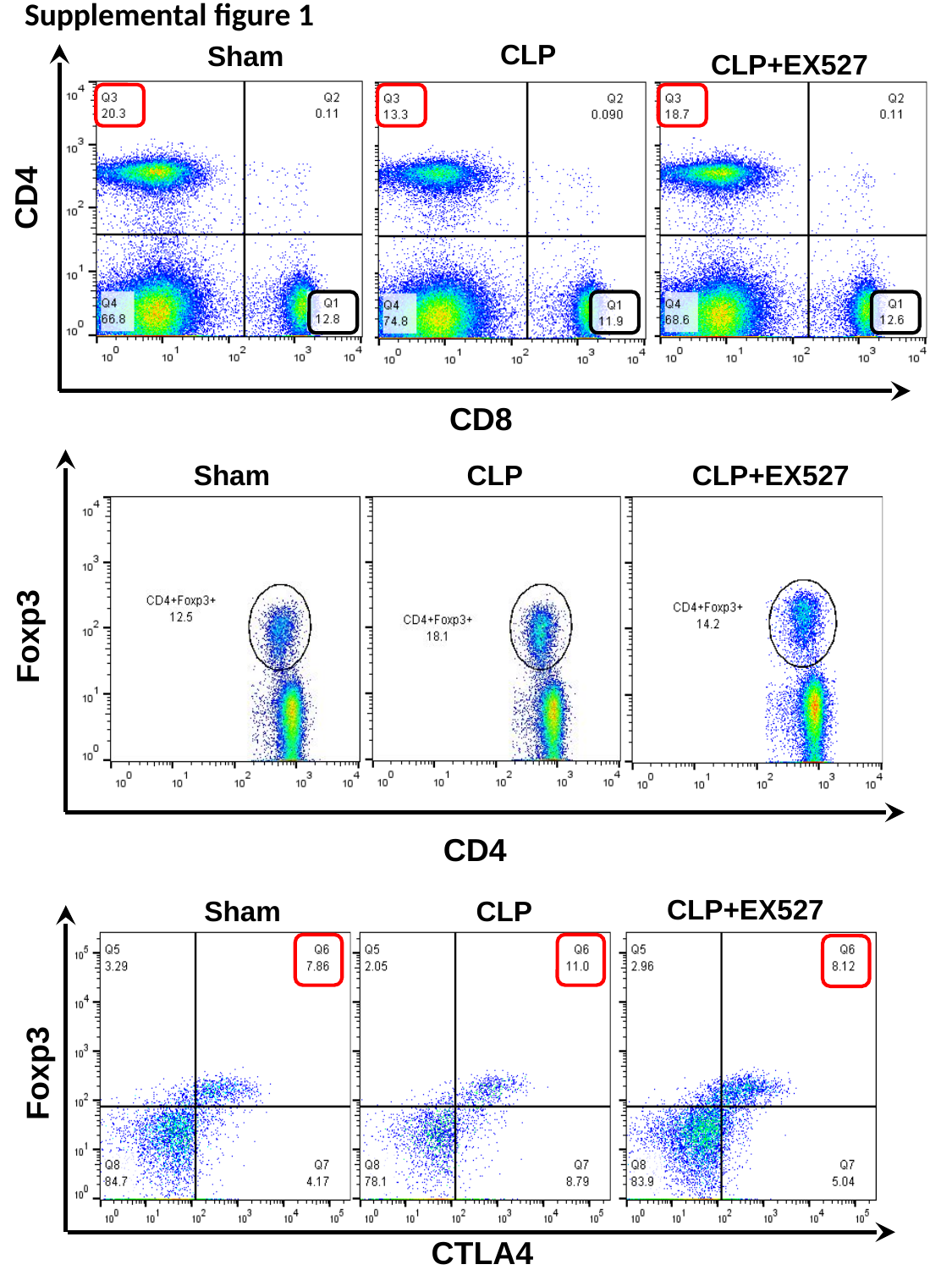

Supplemental figure 1
CLP
Sham
CLP+EX527
CD4
CD8
Foxp3
CD4
Sham
CLP
CLP+EX527
CLP+EX527
Sham
CLP
CTLA4
Foxp3

Supplement: Supplementary 1 — Figure 1: representative flow cytometry data of T cell subpopulations. Splenocytes from sham, sepsis (CLP), and septic mice treated with EX-527 (CLP + EX-527) were analyzed by FACS. Representative FACS data of CD8+ versus CD4+ T cells (A) and Foxp3+ versus CD4+ T cells (B). Gated cells are CD4+Foxp3+. Representative FACS data of CTLA4+ versus Foxp3+ T cells (C). Cells are gated on CD4+. Data are representative of three independent analyses; n = 15 mice/group. [file 2402593.f1.pptx]
