# Supplementary material for: Sirtuin1 Targeting Reverses Innate and Adaptive Immune Tolerance in Septic Mice
Source: J Immunol Res. 2018 Jul 4;2018:2402593. doi: 10.1155/2018/2402593 (PMC6057336; doi:10.1155/2018/2402593)
Supplement: Supplementary 2 — Figure 2: the decrease in CD4+Foxp3+ TReg cells is maintained with SIRT1 inhibition at 48 h and the effect of SIRT1 inhibition on CD4+ reprogramming. FACS analysis CD4+ T cells (A) and CD4+Foxp3+ cells (B) at 12 (n = 5 mice/group), 30 (n = 15 mice/group), or 48 h (n = 5 mice/group) from sham, sepsis (CLP), and septic mice treated with EX-527 (CLP + EX-527). Data expressed as mean ± SEM. (C) Graphic representation of SIRT1 reprogramming CD4+ cells during sepsis. [file 2402593.f2.pptx]

## Slide 1
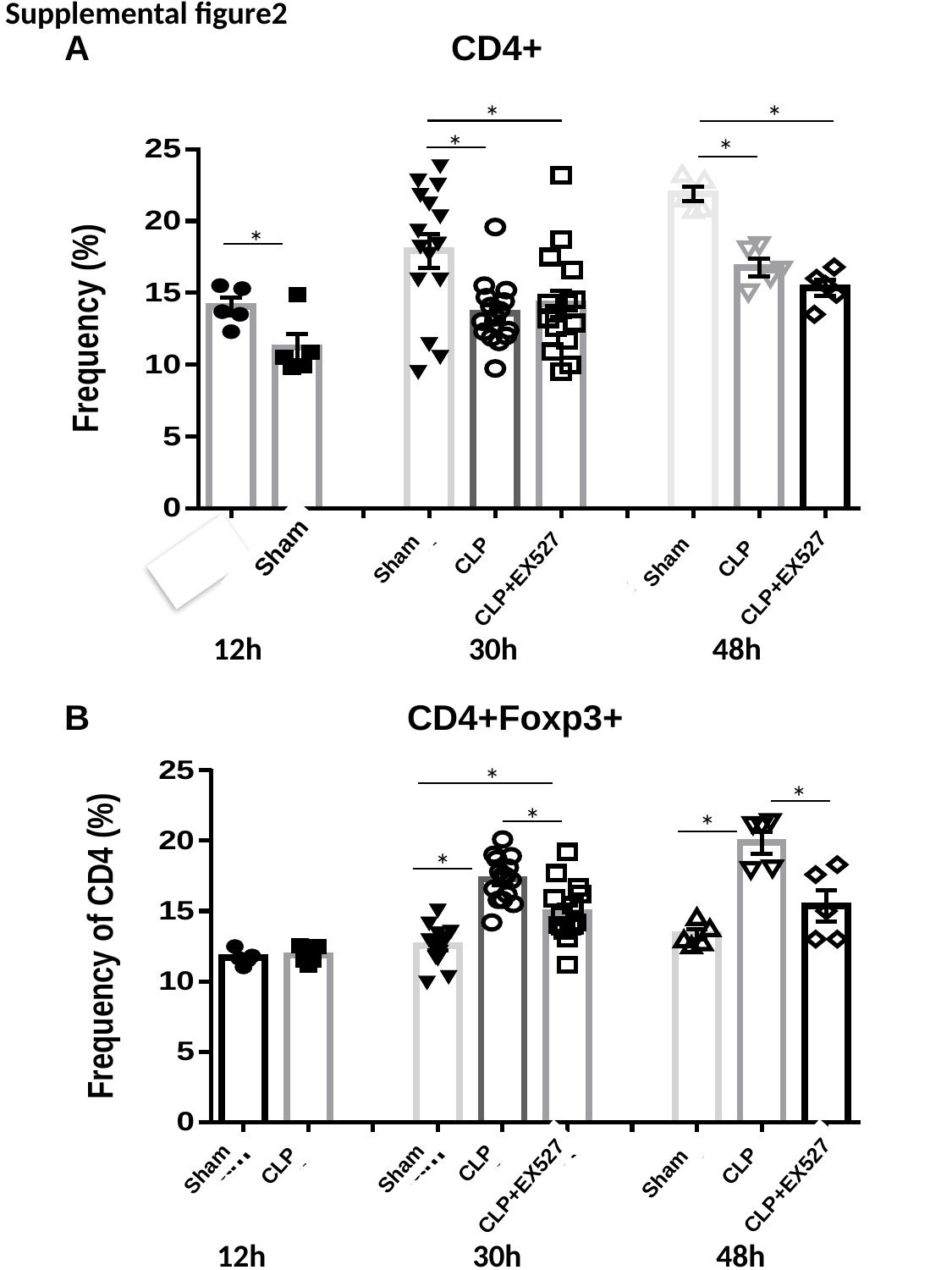

Supplemental figure2
A
CD4+
*
*
*
*
*
CLP
CLP
CLP
Sham
Sham
CLP+EX527
CLP+EX527
12h
30h
48h
B
CD4+Foxp3+
*
*
*
*
*
CLP
CLP
CLP
Sham
Sham
Sham
CLP+EX527
CLP+EX527
12h
30h
48h
Sham
